# Screening of Esophageal Varices by Noninvasive Means in Chronic Liver Disease

**DOI:** 10.5005/jp-journals-10018-1252

**Published:** 2018-05-01

**Authors:** Enayet Hossain, Ferdaus Ahammed, Satyajit K Saha, Syed A Foez, Mohammad A Rahim, Sheikh M Noor-e-Alam, Abu S Abdullah

**Affiliations:** 1 Department of Medicine, Sylhet MAG Osmani Medical College, Sylhet, Bangladesh; 2 Department of Hepatology, Sylhet MAG Osmani Medical College, Sylhet, Bangladesh; 3 Department of Hepatology, Abdul Malek Ukil Medical College, Noakhali, Bangladesh; 4 Department of Hepatology, Bangabandhu Sheikh Mujib Medical University, Dhaka, Bangladesh; 5 Department of Medicine, Moulvibazar District Sadar Hospital, Moulvibazar, Bangladesh

**Keywords:** Chronic liver disease, Esophageal varices, Prognosis, Screening.

## Abstract

**Introduction:**

Noninvasive assessment of esophageal varices (EV) decreases the medical and financial burden related to screening and helps in the management of patients with chronic liver diseases (CLDs). In this study, our aim was to assess the utility of the platelet count/spleen diameter index for the noninvasive evaluation of EV.

**Materials and methods:**

In this cross-sectional observational study, a total of 100 CLD patients underwent screening endoscopy for EV in Medicine and Gastroenterology Department, Sylhet MAG Osmani Medical College Hospital, Sylhet, Bangladesh. Platelet count/spleen diameter ratio was assessed in all patients and its diagnostic implication was calculated.

**Results:**

Upper gastrointestinal endoscopy revealed that 45 (45.0%) patients had medium EV followed by 27 (27.0%) that had small EV and 19 (19.0%) patients had large EV. Receiver operator characteristic (ROC) curve was constructed using platelet count/spleen index, which gave a cut-off value of >905. The validity of platelet count/spleen index evaluation of CLD was: Sensitivity 92.3%, specificity 66.7%, accuracy 90.0%, positive predictive value (PPV) and negative predictive value (NPV) were 96.6 and 46.2% respectively. True positive was 84 cases, false positive 3 cases, false negative 7 cases, and true negative 6 cases. If we consider cut-off value as 909 in the evaluation of EV in CLD, then true positive was 85 cases, false positive 3 cases, false negative 6 cases, and true negative 6 cases. From this, by calculation, sensitivity was 93.4%, specificity 66.7%, accuracy 91%, PPV 96.6%, and NPV 50%.

**Conclusion:**

The platelet count/spleen index may be proposed to be a safe and reliable mean of screening of EV in CLD patients; however, case-control study would be required to validate this.

**How to cite this article:** Hossain E, Ahammed F, Saha SK, Foez SA, Rahim MA, Noor-e-Alam SM, Abdullah AS. Screening of Esophageal Varices by Noninvasive Means in Chronic Liver Disease. Euroasian J Hepato-Gastroenterol 2018;8(1):18-22.

## INTRODUCTION

Chronic liver disease is a major cause of morbidity and mortality throughout the world. Owing to unawareness, lack of facilities, and poverty, underdeveloped countries suffer more. It follows as an indolent course and eventually patients develop the complications of liver decompensation characterized by variceal bleeding from portal hypertension, ascites, hepatorenal syndrome, hepatic encephalopathy, and spontaneous bacterial peritonitis.^1^ Esophageal varices are generally the most common clinical manifestation of portal hypertension, and ruptured varices are the deadliest complication of portal hypertension.^2^ Varices usually form when portal pressure exceeds 10 mm Hg and bleeds when it exceeds 12 mm Hg.

Endoscopic screening for EV is currently recommended in all patients at the time of diagnosis of cirrhosis.^3^ As far as patients with no varices at screening endoscopy are concerned, surveillance should be performed every 2 years on patients with stable liver function and every year on those who show abnormal liver function. Finally, endoscopy should be repeated every year when screening endoscopy reveals small varices.^4^

The average prevalence of EV in cirrhotic patients ranges from 60 to 80%, depending on severity and etiology of liver disease,^5^ and empiric treatment of all cirrhotic patients would expose a significant proportion of treated patients to unnecessary and potentially harmful side effects. Therefore, the use of accurate and specific means that could noninvasively diagnose EV would likely increase the cost/benefit of empiric treatment by decreasing the number of patients who are administered avoidable treatment and by increasing the number of properly screened and treated patients. Such a noninvasive means should have a confident safety profile (i.e., NPV approaching 100%) so as to avoid missing the diagnosis in patients at risk, and a relevant cost/benefit profile so as to avoid unnecessary endoscopy and/ or treatment of patients who would not benefit from therapy (i.e., high PPV).^6^ In this scenario, the use of the platelet count/spleen diameter ratio for the noninvasive assessment of EV seems to have been shown to fulfill these requirements and is based on pathophysiological criteria as well.^7^ Furthermore, the diagnostic accuracy of this parameter in the follow-up of patients free from EV at screening endoscopy has been validated in several studies.^8^ Lastly, preliminary results obtained by other authors demonstrated that the diagnostic accuracy of the platelet count/spleen diameter ratio is maintained in subsets of patients with different etiologies of liver disease and by applying different methodologies, thus suggesting the generalized ability of the diagnostic method.^9,10^ However, little is known about the utility of this procedure in patients with CLD in Bangladesh, although this is a country of 160 million people with moderate levels of prevalence of hepatitis B virus (HBV) and hepatitis C virus (HCV). In Bangladesh, CLD and its complications represent a huge burden of hospital admission. Being a resource-constrained country, the implications of noninvasive diagnosis of EV would be enormous. The study presented here has been accomplished in an academic hospital of Bangladesh to assess the utility of noninvasive diagnosis of EV in CLD patients.

## MATERIALS AND METHODS

This was a cross-sectional observational study. The study was carried out in the Department of Medicine and Department of Gastroenterology, Sylhet MAG Osmani Medical College Hospital, Sylhet, from January 2014 to December 2014. All admitted patients having CLD in the Department of Medicine and Department of Gastroenterology, Sylhet MAG Osmani Medical College Hospital, Sylhet during the study period was the study population and those who fulfilled the inclusion and exclusion criteria was selected sample. Sampling method was purposive sampling.

### Inclusion Criteria

All the patients diagnosed to be suffering from CLD irrespective of etiology, attending the inpatient department of the study place during the study period were enrolled in the study. The exclusion criteria were active bleeding current alcohol intake (patients with alcohol-related liver cirrhosis was included if abstinent for at least 6 months prior to endoscopy), previous endoscopic sclerosis or band ligation of EV, previous surgery for portal hypertension, or transjugular intrahepatic portosystemic stent shunt placement, p-blocker therapy, patients who did not undergo endoscopic evaluation, and patient or eligible attendant unwilling to participate in this study.

## RESULTS

The mean age was 45.11 ± 15.3 years with range from 14 to 80 years. It was observed that almost one-fourth (23.0%) patients belonged to age 50 to 59 years. Regarding sex distribution, majority (84.0%) of patients were males and 16 (16.0%) were females. The etiology of CLD was HBV in 45% of patients and etiology could not be ascertained in 38% patients.

The presenting complaints of the study population indicated that majority (95.0%) patients had swelling of body followed by 64 (64.0%) had yellowish discoloration, 5 (5.0%) had disorientation, 1 (1.0%) vomiting of blood, and 1 (1.0%) had passage of black tarry stool. It was also found that 65 (65.0%) patients had jaundice, 90 (90.0%) had anemia, 9 (9.0%) had clubbing, 96 (96.0%) had edema, 6 (6.0%) had caput medusa, and 6 (6.0%) had flapping tremor. Abdominal examination findings of the study population indicated that 88 (88.0%) patients had ascites. More than half (63.0%) had splenomegaly and 14 (14.0%) had hepatomegaly.

The biochemical parameters of the patients are shown in Table 1.

Mild, moderate, and severe ascites were found in 28, 31, and 30 patients, respectively, with 11 patients with no ascites. The spleen diameter was 131.2 ± 26.5 mm with a range of 90 to 210 mm.

**Table 1:** **Table 1:** Biochemical parameters of the study population

*Biochemical parameters*		*Mean± SD*		*Range (min-max)*	
AST (U/L)		48.5 ± 42.3		(23-432)	
ALT (U/L)		95.5 ± 92.1		(32-784)	
Total bilirubin (mg/dL)		2.5 ± 1.1		(0.7-6.5)	
Serum albumin (g/dL)		2.5 ± 0.4		(1.6-4.0)	
Prothrombin time (s)		20.9 ± 4.1		(15-45)	
Serum creatinine (mg/dL)		1.0 ± 0.2		(0.7-1.7)	
Platelet count (mm^3^)		106,190 ± 45,816		(3,000-252,000)	

AST: Aspartate transaminase; ALT: Alanine transaminase

**Graph 1:**
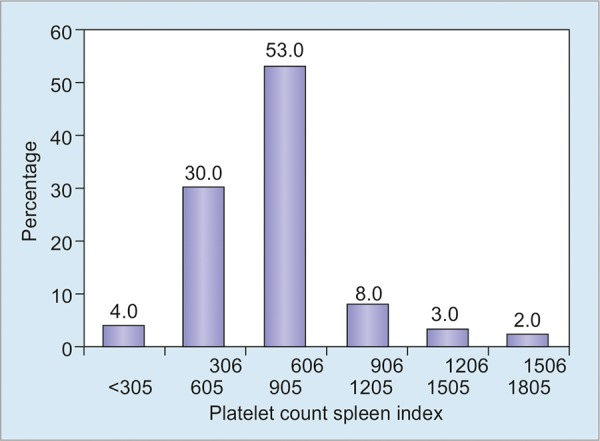
Platelet count *vs* spleen index of the CLD patients

**Graph 2:**
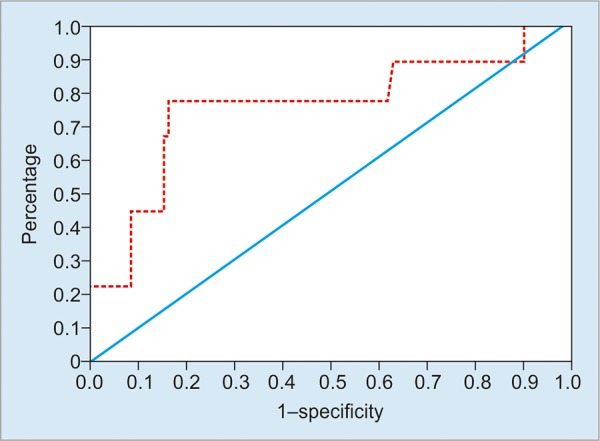
Receiver-operator characteristic curve of platelet count/spleen index for prediction of EV

Endoscopic examination of EV of the study population observed that majority, i.e., 45 (45.0%) patients, had medium EV followed by 27 (27.0%) had small and 19 (19.0%) patients had large EV. Nine (9.0%) patients had no EV. Child-Pugh criteria of the study population revealed that majority (59.0%) had mild presence and degree of ascites, 10 (10.0%) had mild encephalopathy. The mean Child-Pugh score was found to be 10.5 ± 1.5, with range from 6 to 13. The mean platelet count/spleen index was found to be 825.0 ± 353.5, with range from 28 to 1800.

As shown in Graph 1, about half (53.0%) patients had platelet count/spleen index between 606 and 905.

The area under the ROC curves for the EV predictors is shown in Graph 2. Based on the ROC curves, platelet count/spleen index had area under the curve 0.793. Receiver-operator characteristic curve was constructed using platelet count/spleen index, which gave a cut-off value >905, with 92.3% sensitivity and 66.7% specificity for prediction of EV. In platelet count/spleen index if we consider cut-off value as 905 in the evaluation of EV in CLD, true positive was 84 cases, false positive 3 cases, false negative 7 cases, and true negative 6 cases.

In platelet count/spleen index if we consider cut-off value as 909 in the evaluation of EV in CLD, true positive was 85 cases, false positive 3 cases, false negative 6 cases, and true negative 6 cases. From this, by calculation, sensitivity was 93.4%, specificity 66.7%, accuracy 91%, PPV 96.6%, and NPV 50%.

## DISCUSSION

This cross-sectional observational study was carried out to assess if the platelet count/spleen diameter index may be used as an indicator of EV in Bangladeshi patients with EV. The majority of HBV-related patients in this study may be due to increased prevalence of HBV in this area. In this series, majority (95.0%) of patients had swelling of body, followed by 64 (64.0%) had yellowish discoloration, 5 (5.0%) had disorientation, 1 (1.0%) vomiting of blood, and 1 (1.0%) had passage of black tarry stool.

In this study, it was observed that a total of 89 (89.0%) patients had ascites with various degrees. The mean spleen diameter was found to be 131.1 ± 26.5 mm with range from 90 to 210 mm. Umar et al^11^ mentioned that a splenic size of >130 mm was found in 75.0% cases having mean splenic diameter of 145 ± 23.9 mm, which are comparable with the current study.

Majority (45.0%) of patients in the series had medium size EV followed by 27.0% had small and 19.0% patients had large EV. Agha et al^12^ showed 72.0% patients had endoscopic evidence of EV. In this study, EV were found in more patients may be due to late presentation of the patients to the hospital.

In this study, it was observed that the area under the ROC curves for the predictors of EV platelet count/ spleen index had area under the curve 0.793. Receiver-operator characteristic curve was constructed using platelet count/spleen index, which gave a cut-off value >905, with 92.3% sensitivity and 66.7% specificity for prediction of EV. In platelet count/spleen index with cut-off value as 905 in the evaluation of EV in CLD, true positive was 84 cases, false positive 3 cases, false negative 7 cases, and true negative 6 cases. The validity of platelet count/spleen index in evaluation of CLD was sensitivity 92.3%, specificity 66.7%, accuracy 90.0%, PPV and NPV were 96.6 and 46.2% respectively.

Agha et al^12^ found that platelet count/spleen diameter ratio <885 had 100% sensitivity (89-100, 95% confidence interval [CI]), 92% specificity (62-99, 95% CI), 12.0 positive likelihood ratio, and 0.01 negative likelihood ratio for the diagnosis of EV. Accuracy of the platelet count/spleen diameter ratio cut-off value as evaluated by the c-index was 0.989 (95% CI 0.897-0.992).

In this study, if we consider platelet count/spleen index cut-off value as 909 in the evaluation of EV in CLD, true positive was 85 cases, false positive 3 cases, false negative 6 cases, and true negative 6 cases. The validity of platelet count/spleen index evaluation of CLD was sensitivity 93.4%, specificity 66.7%, accuracy 91%, PPV 96.6%, and NPV 50%.

Receiver-operating characteristic curve for platelet/ spleen diameter ratio = 909 was performed by Sarangapani et al^13^ and it was found that area under the curve was: 0.883 95% CI (0.81-0.91)]. The sensitivity and specificity were 88.5 and 83% respectively.

Sarangapani et al^13^ used the platelet count/spleen diameter ratio cut-off determined by Giannini et al^14^ in predicting large varices and found that the NPV of platelet count/spleen diameter ratio = 909 was 100%. Agha et al^15^ studied 114 patients with compensated HCV-related cirrhotics, and 909 cut-off showed NPV of 100% and a PPV of 93.8% for the diagnosis of EV. Baig et al^16^ reported a cut-off value of 1014, which gave PPV and NPV of 95.4 and 95.1% respectively. In Sarangapani et al^13^ study, the cut-off value = 909 had 88.5% sensitivity, 83.0% specificity, 83.5% PPV, and 90.5% NPV for the diagnosis of EV. Similarly, Giannini et al^14^ obtained that the cut-off value = 909 had 91.5% sensitivity (95% CI 85.0-95.9%), 67.0% specificity (95% CI 56.9-76.1%), 76.6% PPV, 87.0% NPV, 2.77 positive likelihood ratio, and 0.13 negative likelihood ratio for the diagnosis of EV. Accuracy of the platelet count/spleen diameter ratio was maintained for both severity and etiology of disease subgroups. Considering the entire cohort of patients, the accuracy (area under the ROC curve or c-index) of the platelet count/spleen diameter ratio for the diagnosis of EV was 0.860 (standard error 0.026, 95% CI 0.807-0.904). In particular, the diagnostic accuracy of the platelet count/ spleen diameter ratio for EV was significantly greater as compared with either accuracy of platelet count alone *(c*-index = 0.836, standard error 0.028, 95% CI 0.7800.883). Difference between areas = 0.024, standard error 0.012, 95% CI 0.001-0.048, p < 0.05 or accuracy of spleen diameter alone (c-index = 0.802, standard error 0.029, 95% CI 0.743-0.853). Difference between areas = 0.058, standard error 0.025, 95% CI 0.010-0.107, p < 0.05. A platelet count/spleen diameter ratio cut-off of 909 (N/mm^3^)/mm had 91.5% sensitivity (95% CI 85.0-95.9%), 67.0% specificity (95% CI 56.9-76.1%), 76.6% PPV, 87.0% NPV, 2.77 positive likelihood ratio, and 0.13 negative likelihood ratio for the diagnosis of EV. Their results showed a plot of both PPV and NPV of the platelet count/spleen diameter cut-off ratio = 909, considering various prevalence of disease. The diagnostic efficiency (true positives + true negatives/N) of the platelet count/spleen diameter cut-off ratio = 909 was 80.3% (95% CI 74.4-85.3%). With regards to patients with viral etiology of cirrhosis, a platelet count/spleen diameter ratio cut-off of 909 had 83.9% accuracy for the diagnosis of EV (95% CI 76.5-89.7%), while accuracy was 95.9% in patients with alcohol-induced cirrhosis (95% CI 84.6-99.5%) and 89.5% in patients with primary biliary cirrosis (PBC) (95% CI 74.4-97.2%). Their results showed that the sensitivity of the platelet count/spleen diameter ratio cut-off 909 in patients with various etiologies of liver disease was above 80% in all subgroups, while specificity was lower in patients with virus induced cirrhosis as compared with both alcoholic and PBC patients. Noteworthy, both PPV and NPV were high in all the subgroups of patients.

## CONCLUSION

This study was undertaken to assess noninvasively EV by platelet count/spleen index in CLD. Chronic liver disease is more common in fifth and sixth decade and male predominant. Hepatitis B virus was the commonest etiology of cirrhosis and swelling of body and yellowish discoloration were the frequent presenting complaints. Jaundice, anemia, edema, ascites, and splenomegaly were more common. The use of the platelet count/spleen index safely identified patients without EV and allowed us to identify a large number of patients with EV. Applying the platelet count/spleen index could be proposed in clinical practice as part of the diagnostic workup of cirrhotic patients in order to decrease the medical and financial burden of the endoscopy to EV screening and follow-up.
